# RPS19 and RPL5 Haploinsufficient Models Reveal Divergent Ribosomal Subunit Controls of Fetal Hematopoiesis

**DOI:** 10.21203/rs.3.rs-7563799/v1

**Published:** 2025-09-26

**Authors:** Lionel Blanc, Yuefeng Tang, Te Ling, Rashid Mehmood, Alexis Bertrand, Julien Papoin, Mushran Khan, Riaz Rao, Jianing Xu, Vincent Schulz, James Palis, Laurie Steiner, Betsy Barnes, YongRui Zou, Philippe Marambaud, Robert Signer, Irene Roberts, Deena Iskander, Leonard Zon, Senthil Bhoopalan, Mitchell Weiss, Jeffrey Lipton, Patrick Gallagher, Narla Mohandas, Naomi Taylor, Sebastien Durand, John Crispino

**Affiliations:** Feinstein Institute For Medical Research; Northwell; St. Jude Children’s Research Hospital; Department of Hematology, St. Jude Children’s Research Hospital; Northwell; Northwell; Northwell; Northwell; Fudan University; Yale University; University of Rochester Medical Center; University of Rochester; Northwell; Northwell; Feinstein Institute for Medical Research; University of San Diego; University of Oxford; Imperial College London; Boston Children’s Hospital; St Jude; St. Jude Children’s Research Hospital; Northwell; Nationwide; New York Blood Center; Institut de Génétique Moléculaire de Montpellier, CNRS; CRCL; St Jude Children’s Research Hospital

## Abstract

Diamond Blackfan anemia syndrome (DBAS) is a congenital ribosomopathy caused by haploinsufficiency of ribosomal proteins (RPs), but how RP stoichiometry and activity regulates erythroid development remains enigmatic. Using novel *in vivo* models, we uncover strikingly divergent functions for the small and large ribosomal subunit proteins RPS19 and RPL5 in fetal hematopoiesis. While RPL5 haploinsufficiency causes hematopoietic stem and progenitor cell (HSPC) accumulation and prenatal lethality via p53-mediated ferroptosis of mature erythroid progenitors, RPS19 haploinsufficiency leads to HSPC depletion and impaired erythroid expansion through p53-dependent apoptosis. The latter is accompanied by translational and transcriptional dysregulation, including the upregulation of *RUNX1,* which is also observed in RPS- haploinsufficient DBAS patients. Importantly, *Runx1* deletion in RPS19-haploinsufficient mice partially rescues HSPC numbers. These findings reveal subunit-specific RP functions in controlling fetal hematopoiesis and demonstrate how imbalanced RP stoichiometry disrupts developmental programs, providing crucial mechanistic insights into DBAS pathogenesis and the basis for its clinical heterogeneity.

Dysregulation of translational control occurs in numerous contexts including cell growth and development, aging, immunity, and responses to environmental changes and may contribute to malignancy, inflammation, autoimmune disease, and other disorders^1–3^. Ribosomopathies are a group of inherited disorders associated with ribosomal protein haploinsufficiency or defects in ribosome biogenesis^4^. While all cells require ribosomes, ribosomopathies often demonstrate tissue-specific defects, sometimes with increased cancer susceptibility^5^. Inherited ribosomopathies with hematologic features include Diamond Blackfan anemia syndrome (DBAS), Schwachman Diamond syndrome (SDS), and X-linked dyskeratosis congenita, among others^6^. DBAS, the archetype of ribosomopathy^4^, is a congenital bone marrow failure syndrome commonly associated with mutations or deletions of ribosomal genes, leading to ribosomal haploinsufficiency, resulting in nucleolar stress and p53 activation^7^. While p53 inhibition is known to ameliorate anemia in DBAS, the precise underlying mechanisms remain poorly understood^8^.

Ribosomopathies remain challenging to study within the hematopoietic system, and few animal models have successfully recapitulated the diverse clinical features of the diseases^9,10^. In the context of DBAS, previous attempts to generate animal models with germline deletions of ribosomal proteins^11,12^ revealed that complete knockout led to pre-implantation embryonic lethality, whereas heterozygous RP deletion resulted in minimal or no noticeable phenotype. Conditional knockout mouse models, under constitutive or inducible promoters, have been generated and display some hematopoietic phenotypes upon RP deletion^9,13^. However, these models do not present reduced reticulocyte counts likely due to compensatory splenic stress erythropoiesis, a process that does not appear to occur in humans^14^. Clinically, DBAS usually manifests soon after birth and is characterized by macrocytosis, reticulocytopenia and a scarcity of erythroid precursors in the bone marrow^15^. The presence of other hematopoietic lineage defects in certain patients suggests a broader underlying defect in hematopoietic stem and/or progenitor cells (HSPCs)^16,17^. However, these aspects remain understudied, and it is unclear whether they are a universal consequence of RP mutations or whether there are differences specific to mutations in large versus small subunits. Thus, DBAS presents a unique framework to uncover novel pathways by which ribosomal subunits regulate hematopoiesis.

To investigate how specific RP subunits contribute to this process, we generated *in vivo* mouse models of RPS19 and RPL5 haploinsufficiency and performed mechanistic studies during fetal development. Our findings reveal distinct, subunit-specific roles for RPS19 and RPL5 in fetal hematopoiesis, uncovering specific roles of these RPs in fetal hematopoiesis. These results demonstrate new physiological and divergent functions for ribosomal proteins beyond their canonical role in translation and provide insights into how disrupted RP stoichiometry alters developmental hematopoiesis and contributes to disease pathogenesis.

## RESULTS

### Monoallelic deletion of Rps19 or Rpl5 in hematopoietic cells phenocopies clinical characteristics of DBAS patients.

**Figure 1:**
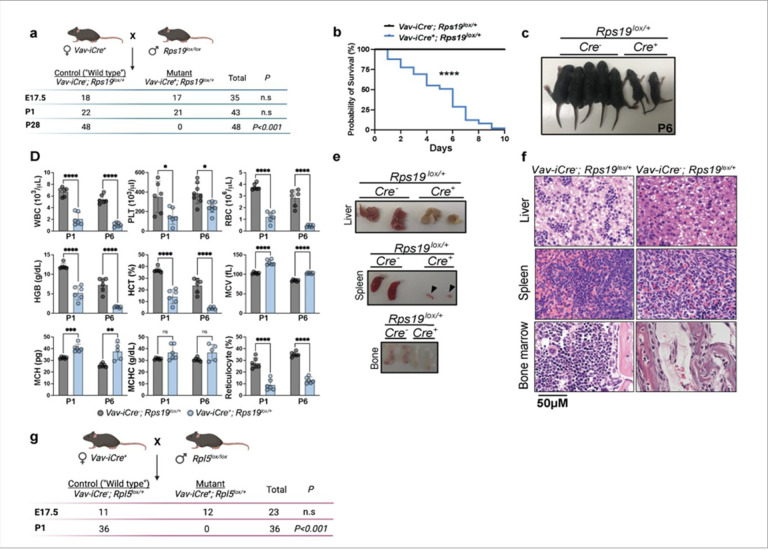
Deletion of one copy of *Rps19* or *Rpl5* leads to severe hematopoietic defects at birth. (a) Genotype counts from *Rps19*^*lox/+*^ intercrossing. (b) Survival curves from *Rps19*^*lox/+*^ mice. (c) Photos of control (*Cre*^−^) and mutant (*Cre*^+^) *Rps19*^*lox/+*^ 6 days after birth (P6). (d) Complete blood counts at P1 and P6. Upper panel: white blood cells (WBC), platelets (PLT), red blood cells (RBC). Middle panel: hemoglobin (HGB), hematocrit (HCT), mean corpuscular volume (MCV). Lower panel: mean corpuscular hemoglobin (MCH), mean corpuscular hemoglobin concentration (MCHC), reticulocytes. (e) Images of the liver, spleen and bone marrow at P6 in control and mutant *Rps19*^*lox/+*^ at P6. The arrow denotes the mutant spleen. (f) Light microscopy images (hematoxylin and eosin) highlighting the architecture and cellular composition of the same hematopoietic organs in control and mutant *Rps19*^*lox/+*^ mice at P6. (g) Genotype counts from *Rpl5*^*lox/+*^ mice. All data are presented as mean ± standard deviation (n.s.: not significant, *p<0.05, **p<0.01, ***p<0.001, ****p<0.0001).

To gain insights into the role of RPs during hematopoiesis, we generated two conditional mouse models using CRISPR-Cas9 editing to delete specific regions of the *Rps19* and *Rpl5* genes— two of the most frequently mutated RP in DBAS (**Figure S1a and b**). The use of the *Vav-Cre* promoter restricts the Cre activity to the hematopoietic system beginning at midgestation^18,19^. We noticed that, while there was no *in utero* loss of *Rps19*^*lox/+*^ fetuses, pups did not reach weaning age (Fig. 1a). Survival analyses demonstrated that *Vav-Cre*^*+*^; *Rps19*^*lox/+*^ mice (hereafter referred to as *Rps19*^lox/+^) began dying after birth and were all dead by postnatal day 10 (P10) (Fig. 1b). These mice were paler and smaller (Fig. 1c) and complete blood counts revealed pancytopenia suggestive of bone marrow failure (Fig. 1d). Analysis of red cell indices revealed a significant increase in their mean cell volume (MCV), which was not due to reticulocytosis, since the reticulocyte count was dramatically reduced, in contrast to previous models (Fig. 1d).

In mice, the spleen assumes a hematopoietic function from birth and during periods of hematopoietic stress, especially during erythroid stress^14^. Notably, the spleens of *Rps19*^*lox/+*^ mice were atrophic (Fig. 1e, black arrows). Further, gross and histological analyses of hematopoietic tissues^20^ revealed a severely hypocellular bone marrow and an absence of red cell precursors in both the liver and spleen of *Rps19*^*lox/+*^ at P6 (Fig. 1e, f).

In contrast, heterozygous deletion of *Rpl5* under the *Vav* promoter led to perinatal lethality (Fig. 1g). *Vav-Cre*^*+*^; *Rpl5*^*lox/+*^ embryos (hereafter referred to as *Rpl5*^*lox/+*^) were detected at Mendelian ratios at E17.5 but none survived to birth, suggesting that *Rpl5* heterozygosity results in late- gestation lethality (Fig. 1g). Together, these models provide a valuable resource to address critical, mechanistic, questions related to the role of RP in fetal hematopoiesis.

### Haploinsufficiency of RPS19 or RPL5 disrupts fetal erythropoiesis through divergent effects on erythroid progenitors.

**Figure 2:**
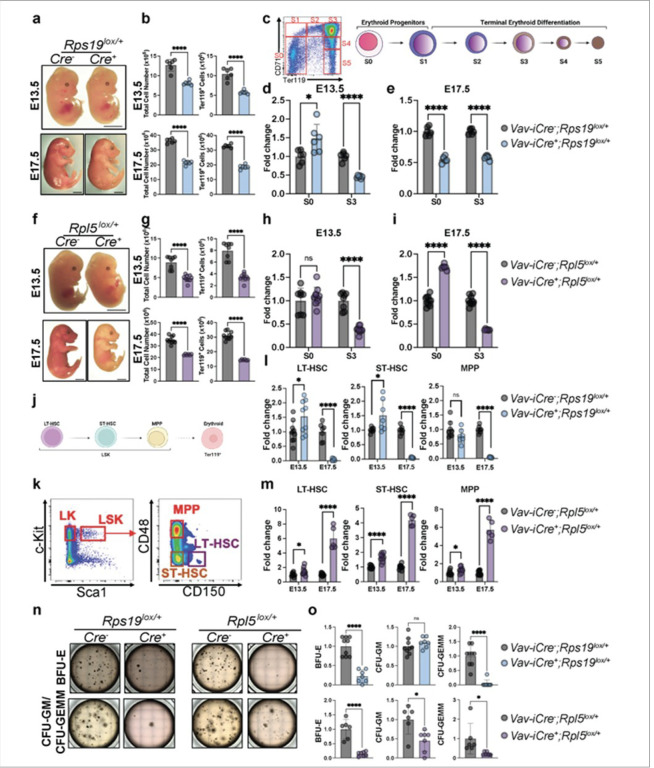
Deletion of one allele of *Rps19* or *Rpl5* leads to divergent effects on the hematopoietic stem and progenitor cell (HSPC) compartment during fetal hematopoiesis. (a) Images of E13.5 (upper) and E17.5 (lower) control and *Rps19*^*lox/+*^ embryos. (b) Fetal liver (FL) cellularity and Ter119^+^ counts in E13.5 (upper) and E17.5 (lower) control and *Rps19*^*lox/+*^ embryos. (c) Gating strategy to assess erythropoiesis in the FL. (d) Quantification of cells in S0 and S3 in E13.5 control, and *Rps19*^*lox/+*^ embryos expressed as a fold change relative to control. (e) Quantification of cells in S0 and S3 in E17.5 control, and *Rps19*^*lox/+*^ embryos expressed as a fold change relative to control. (f) Images of E13.5 (upper) and E17.5 (lower) control and *Rpl5*^*lox/+*^ embryos. (g) FL cellularity and Ter119+ counts in E13.5 (upper) and E17.5 (lower) control and *Rpl5*^*lox/+*^embryos. (h) Quantification of cells in S0 and S3 in E13.5 control, and *Rpl5*^*lox/+*^ embryos expressed as a fold change relative to control. (i) Quantification of cells in S0 and S3 in E17.5 control, and *Rpl5*^*lox/+*^ embryos expressed as a fold change relative to control. (j) Schematic representation of the HSPC populations used in the study. (k) Gating strategy to assess early hematopoiesis in the FL. (l) Quantification of the different HSPC populations in E13.5 and E17.5 control and *Rps19*^*lox/+*^ embryos expressed as a fold change relative to control. (m) Quantification of the different HSPC populations in E13.5 and E17.5 control and *Rpl5*^*lox/+*^ embryos expressed as a fold change relative to control. (n) Representative images of colony-forming assays performed in E13.5 control, *Rps19*^*lox/+*^ and *Rpl5*^*lox/+*^ embryos. BFU-E: burst-forming unit-erythroid, CFU-GM: colony-forming unit- granulocyte/macrophage, CFU-GEMM:colony-forming unit-granulocyte, erythrocyte, monocyte and macrophage. (o) Quantification of colonies obtained from E13.5 control, *Rps19*^*lox/+*^ and *Rpl5*^*lox/+*^ embryos. All data are presented as mean ± standard deviation (*p<0.05, **p<0.01, ***p<0.001, ****p<0.0001).

To understand the striking phenotypic differences observed in our *Rps19* and *Rpl5* DBAS models, we studied hematopoiesis in wild-type (WT) and heterozygous mouse embryos. Immunoblot analyses confirmed that heterozygous deletion of *Rps19* and *Rpl5* resulted in an approximately 50% reduction in their respective protein levels in fetal liver cells (**Figure S2a**). *Rps19*^*lox/+*^ embryos at E13.5 and E17.5 were slightly paler compared to their WT littermates. This was associated with a ~ 50% decrease in both total and red cell progenitor (Ter119^+^) cell numbers, underpinning anemia (Fig. 2a, b). Flow cytometry assays—using CD71 and Ter119 markers to quantify distinct stages of fetal erythropoiesis (Fig. 2c)^21^—revealed an accumulation of early erythroid progenitors (S0) at E13.5 (Fig. 2d, S2b, c). Conversely, this same progenitor population was significantly reduced at E17.5 (Fig. 2e). Erythropoiesis was not completely blocked, however, as red cells (S5) were produced, albeit in reduced numbers (**Figure S2b-d**). Together, these results strongly suggest that RPS19 haploinsufficiency causes anemia during fetal life by impairing erythroid progenitor development.

In contrast, *Rpl5*^*lox/+*^ embryos revealed a more severe phenotype. At E13.5 the embryos were noticeably paler than WT littermates and by E17.5, the embryos were smaller and severely anemic (Fig. 2f). Similar to *Rps19*^*lox/+*^*, Rpl5*^*lox/+*^ embryos displayed a 2-fold reduction in total fetal liver cellularity, with a similar reduction in Ter119 + cells (Fig. 2g). At E13.5, a decrease in the S3 erythroid progenitor population was also observed in *Rpl5*^*lox/+*^ embryos (Figs. 2h, S2e, f). Notably though, the impact of *Rps19* and *Rpl5* haploinsufficiency on more primitive Ter119^−^ progenitors (S0) differed markedly by day E17.5. While this S0 population was significantly decreased in *Rps19*^*lox/+*^ embryos, it was dramatically increased in *Rpl5*^*lox/+*^ embryos (Figs. 2h, i, S2g). Indeed, in *Rpl5*^*lox/+*^ embryos, the effects were largely restricted to Ter119^+^ S3 erythroid cells. Taken together, these data demonstrate that while both RPS19 and RPL5 haploinsufficiency lead to decreased red blood cell production during fetal erythropoiesis, the underlying mechanisms leading to erythroid failure differ. Specifically, the loss of early erythroid progenitors by E17.5 is unique to *Rps19*^*lox/+*^ and not observed in *Rpl5*^*lox/+*^ embryos.

### RPS19 haploinsufficiency leads to depletion of the HSPC compartment while RPL5 haploinsufficiency leads to its expansion.

Based on our characterization of these mouse models, and the observation that *Rps19*^*lox/+*^ mice present at birth with pancytopenia (Fig. 1d), we hypothesized that *Rps19* and *Rpl5* haploinsufficiency leads to disparate defects on the fetal HSPC compartment. To test this hypothesis, we quantified the different HSPC populations at E13.5 and E17.5 using commonly used cell surface markers^22^(Fig. 2j, k). Within the lineage-negative (Lin-) population, the LSK (Lin^−^Sca1^+^c-Kit^+^) compartment can be divided into four HSPC subpopulations; the most primitive LT-HSC (CD150^+^CD48^−^LSK) can produce all hematopoietic cell types following transplantation for > 16 weeks whereas ST-HSC (CD150^−^CD48^−^LSK) regenerate hematopoietic cells only transiently and MPP (CD48^+^LSK) represent lineage-biased subsets^23^. While at E13.5, significantly higher levels of LT-HSC and ST-HSC were detected in both *Rps19*^*lox/+*^ and *Rpl5*^*lox/+*^ embryos, relative to WT embryos (Fig. 2l and 2m), by E17.5, there was a dramatic loss of LT-HSC, and ST-HSC in *Rps19*^*lox/+*^ embryos to almost undetectable levels (Fig. 2l). In marked contrast, LT- HSC, and ST-HSC were increased by 4–6-fold in *Rpl5*^*lox/+*^ embryos by E17.5, relative to their WT counterparts (Fig. 2m). These data indicate that *Rps19* but not *Rpl5* haploinsufficiency is deleterious to HSPCs. Furthermore, these data suggest that *Rpl5*^*lox/+*^ HSPCs expand, likely to try and compensate for the severe erythropoietic defects.

However, despite increased numbers, these progenitors were markedly defective in their ability to generate colony forming units (CFU) *in vitro*; the most severe defect was detected in the potential of these progenitors to form erythroid (BFU-E) as compared to myeloid (CFU-GM) colonies (Fig. 2n, o). Together, these results demonstrate specific requirements for RPS19 and RPL5 in shaping the HSC and progenitor compartments during fetal development.

### Increased translation in fetal HSPCs in the context of ribosomal haploinsufficiency.

**Figure 3:**
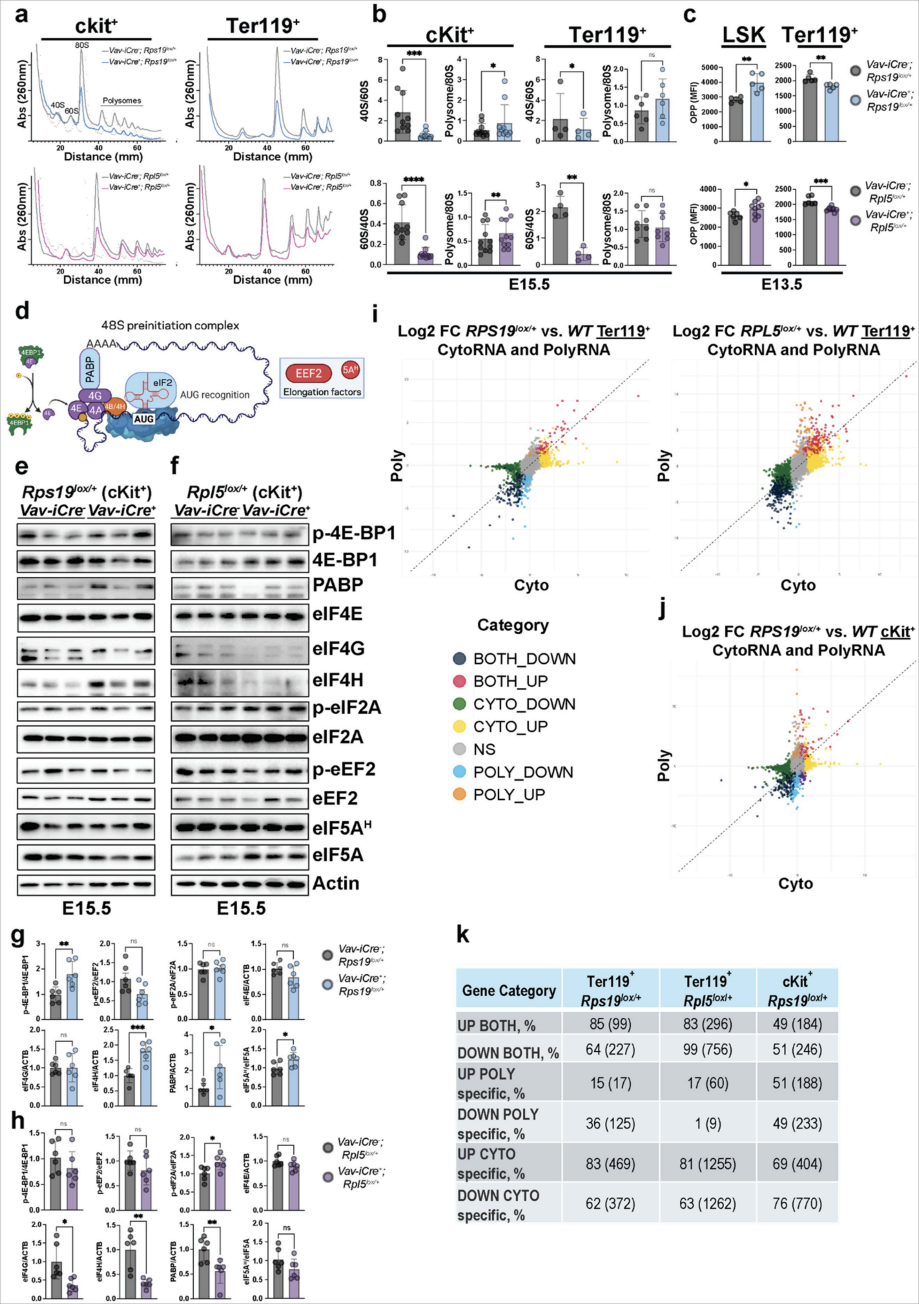
Impaired ribosome biogenesis leads to increased translation in HSPC. (a) Polysome profiles from ckit^+^ and Ter119^+^ cell populations in E15.5 control, *Rps19*^*lox/+*^ or *Rpl5*^*lox/+*^ embryos. (b) Quantification of the 40S and 60S ribosomal subunits, monosomes (80S) and polysomes expressed as ratio from ckit^+^ and Ter119^+^ cell populations in E15.5 control, *Rps19*^*lox/+*^ or *Rpl5*^*lox/+*^ embryos. (c) Quantification of global translation of LSK and Ter119^+^ populations as measured by OPP in E13.5 control, *Rps19*^*lox/+*^ or *Rpl5*^*lox/+*^ embryos. (d) Schematics of the main initiation and elongation factors involved in eukaryotic translation. (e) Western blot analysis of regulators of translation in FL-derived ckit^+^ cells from E15.5 control versus *Rps19*^*lox/+*^ embryos. (f) Western blot analysis of regulators of translation in FL-derived ckit^+^ cells from E15.5 control versus *Rpl5*^*lox/+*^ embryos. (g) Quantification of the western blots for the regulators of translation in FL-derived ckit^+^ cells from E15.5 control versus *Rps19*^*lox/+*^ embryos. (h) Quantification of the western blots for the regulators of translation in FL-derived ckit^+^ cells from E15.5 control versus *Rpl5*^*lox/+*^ embryos. (i) Differential Expression (DE) analyses on the whole cytoplasmic (CYTO) lysate (transcription) and polysomal (POLY) fractions (translation) from Ter119^+^ cells in E15.5 control, *Rps19*^*lox/+*^ or *Rpl5*^*lox/+*^ embryos. (j) Differential Expression (DE) analyses on the whole cytoplasmic (CYTO) lysate (transcription) and polysomal (POLY) fractions (translation) from cKit^+^ HSPCs in E15.5 control, *Rps19*^*lox/+*^ embryos. (k) Quantification of genes up or down regulated at the transcriptional and/or translational level in cKit^+^ and Ter119^+^ populations at E15.5. % of total is shown, with absolute number of differential genes in parentheses. All data are presented as mean ± standard deviation (*p<0.05, **p<0.01, ***p<0.001, ****p<0.0001).

The disparate roles of *Rps19* and *Rpl5* in supporting HSPC maintenance—with a loss of progenitors in the former and an accumulation of functionally abnormal progenitors in the latter, raised the question of whether this might reflect differential effects of small versus large ribosomal subunit deficiency on RP stoichiometry and protein synthesis. As RPs assemble into functional ribosomal subunits to sustain mRNA translation, we performed polysome profiling experiments to analyze 40S and 60S abundance (Fig. 3a). Due to the limited numbers of HSPCs, we performed polysome profiling in the c-Kit^+^ HSPCs. *Rps19*^*lox/+*^ and *Rpl5*^*lox/+*^ progenitors revealed a decrease in the 40S and 60S fractions, respectively (Fig. 3a, b). Despite this defect in ribosome biogenesis, it was surprising to detect an increase in the ratio of polysome/80S in *Rps19*^*lox/+*^ and *Rpl5*^*lox/+*^ c-Kit^+^ HSPCs but not in Ter119^+^ erythroblasts. These data suggest a potential increase mRNA translation specifically in c-Kit^+^ progenitors (Fig. 3a, b).

We therefore directly measured global protein synthesis in E13.5 HSPCs and Ter119 + cells, as a function of o-propargyl-puromycin (OPP) incorporation^24^. Surprisingly, OPP incorporation was significantly higher in *Rps19*^*lox/+*^ and *Rpl5*^*lox/+*^ LSK populations compared to control littermates. Interestingly, the magnitude of the increase was substantially (significantly?) greater in *Rps19*^*lox/+*^ LSK cells as compared to *Rpl5*^*lox/+*^ LSK cells. Increased protein synthesis is not well tolerated by adult HSCs and impairs their function and self-renewal. These data suggest that fetal HSCs, which have higher protein synthesis, may partially tolerate elevated protein synthesis rates in the context of *Rpl5*^*lox/+*^ haploinsufficency, but the magnitude of the effect in *Rps19*^lox/+^ could contribute to HSC depletion. In contrast to LSK cells, OPP incorporation was significantly reduced in Ter119^+^ erythroblasts from both *Rps19*^*lox/+*^ and *Rpl5*^*lox/+*^ mice (Fig. 3c, **Figure S3a**). These data indicate that HSPCs, but not erythroid cells, can compensate for the loss of ribosomes by increasing protein synthesis. This suggests that HSPCs have excess ribosome capacity, but that ribosomes are more limiting for translational activity in erythroid lineage cells.

To gain additional mechanistic insight into the compensatory increase in HSPC protein synthesis, we performed western blot analyses of several key factors involved in translation (Fig. 3d) at E15.5 and E17.5. The expression of initiation factors involved in CAP-dependent translation^25^ were increased (eIF4H) or unaffected (eIF4E, eIF4G) in the *Rps19*^*lox/+*^ c-kit^+^ population at E15.5 and E17.5 (Fig. 3e, g, **Figure S3b, d**) while they were significantly decreased in *Rpl5*^*lox/+*^ c- kit^+^ HSPC (Fig. 3f, h, **Figure S3c, e**). Interestingly, a transient increase was observed at E15.5 in the ratio p-eIF2a /eIF2a in the *Rpl5*^*lox/+*^ model compared to their littermate controls, suggesting activation of the integrated stress response^26^ (Fig. 3f, h, **Figure S3c, e**).

Recently, eIF5a and its post-translational hypusination, emerged as a regulator of human erythropoiesis^27^. Hypusinated-eIF5a (eIF5a^H^) is involved in ribosome functions, preventing ribosome stalling in translation initiation, elongation and termination^28–30^. We previously showed that defects in ribosome biogenesis led to ineffective erythropoiesis in cellular models of ribosomopathies^27,31^. Therefore, we measured the expression levels of eIF5a and eIF5a^H^ in our models. The ratio of eIF5a^H^/eIF5a was specifically elevated in c-kit + HSPC from *Rps19*^*lox/+*^ mice but not in c-kit + HSPC from *Rpl5*^lox/+^ mice compared to littermate controls at E15.5. In addition, the eIF5a^H^/eIF5a ratio was subsequently reduced at E.17.5 in the *Rpl5*^*lox/+*^ mice supporting a specific role for eIF5a^H^ depending on the RP mutated (Fig. 3e–h, **Figure S3b-e**). Together, these data suggest that HSPCs attempt to compensate for the ribosomal defects by enhancing translation. However, this compensation may be maladaptive and drive HSPC depletion.

### Translational alterations in Ter119^+^ cells are primarily a consequence of transcriptional changes.

These data led us to hypothesize that the translational activity would be differentially affected depending on which RP subunit was deleted. To address this, we performed polysome sequencing, which provides insights into translation efficiency by analyzing the distribution of mRNAs across actively translating ribosomal fractions (polysomes)^32^, on c-kit^+^ and Ter119^+^ populations from RPS19 and RPL5 haploinsufficient embryos at E15.5. Differential expression (DE) analyses of whole cytoplasmic lysates (transcription) and polysomal fractions (translation) revealed that RPL5 and RPS19 haploinsufficiency in the Ter119^+^ population primarily caused transcriptional changes (Fig. 3i). Specifically, most mRNAs in Ter119^+^ cells exhibited concordant changes in both transcription and translation (Both up/down category) or the transcriptional changes in these mRNAs did not affect translation (Cyto up/down category): 83% and 85% of the up-translated mRNAs displayed a corresponding increase in transcription in RPL5 and RPS19 haploinsufficient Ter119^+^ cells, respectively (Fig. 3k). Furthermore, the majority of the transcriptional changes did not appear to impact translation in these cells as 81% and 83% of the up-transcribed mRNAs showed no corresponding changes in translation. In striking contrast, in c-kit + HSPC, there was a higher proportion of mRNAs where translational changes occurred independently of transcriptional changes (Poly up/down category) (Fig. 3j–k).

Indeed, 50% of the up-translated mRNAs did not display a similar variation in transcript levels. This strongly suggests that translation efficiency is more severely affected in c-kit^+^ HSPC than in more mature Ter119^+^ erythroid cells. We confirmed these observations by determining the Log2 of the ratio between ti(translational changes) and ti(transcriptional changes) (Log2 ti(translation)/ti(transcription)). The Log2 ti(translation)/ti(transcription) is increased in *Rps19*^*lox/+*^ c-kit^+^ compared to *Rps19*^*lox/+*^ and *Rpl5*^*lox/+*^ Ter119^+^ (**Figure S4**), confirming a higher variation of translation changes in c-kit^+^ compared to Ter119^+^ cells. Unfortunately, we were unable to isolate enough RNA in the polysome fractions from c-kit^+^ cells from RPL5 mutants in order to determine the effect of the haploinsufficiency on translation in these cells. Altogether, these results suggest that RPS19 haploinsufficiency preferentially causes translational alterations in HSPCs while alterations in Ter119^+^ are primarily a consequence of transcriptional changes.

### Transcription is preferentially disrupted in Rps19 haploinsufficient HSPCs.

**Figure 4:**
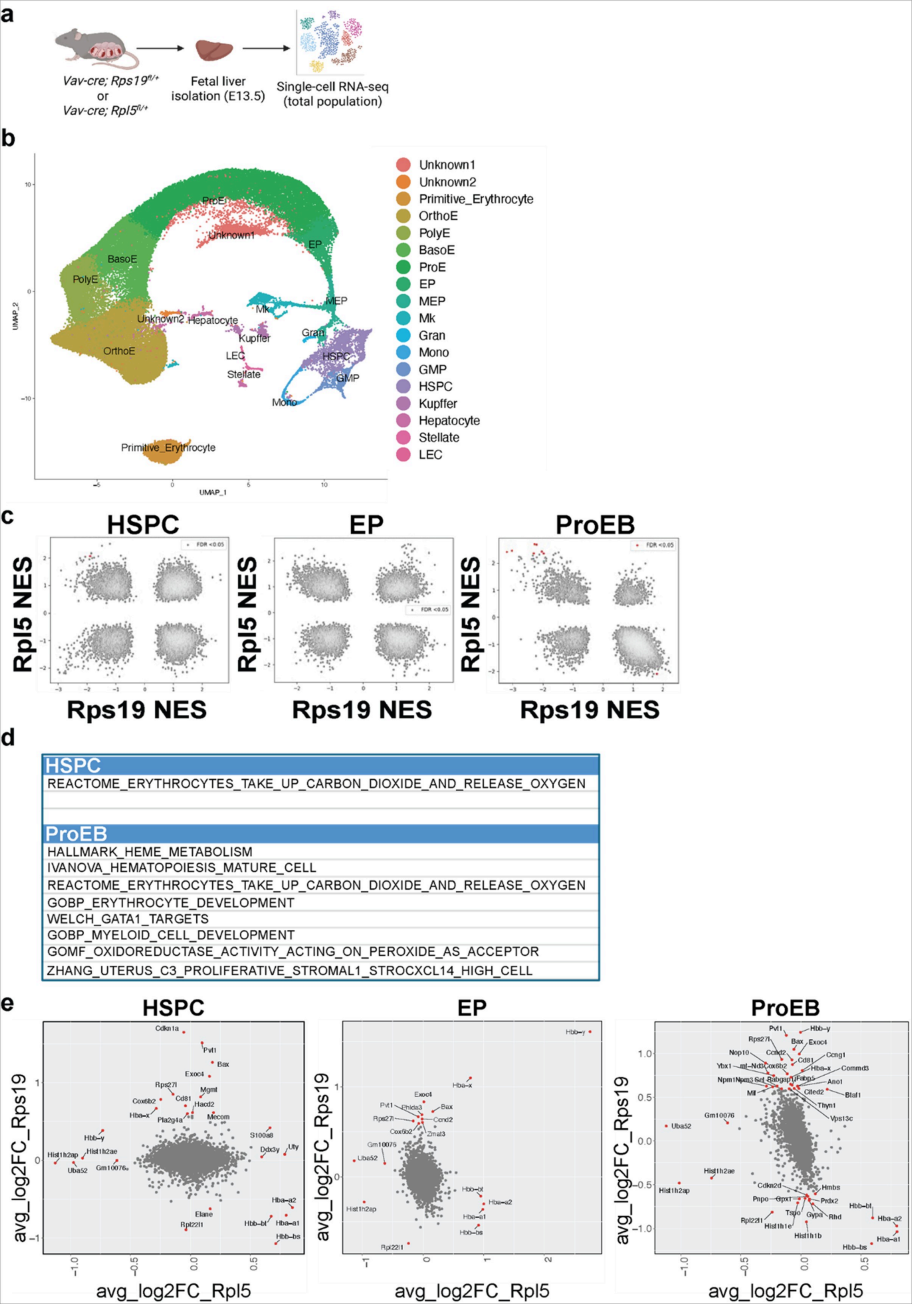
scRNAseq analyses unravel global transcriptional defects in *Rps19*^*lox/+*^ mice. (a) Experimental design for the scRNAseq (10x Genomics) that was performed on total fetal liver cells from E13.5 embryos. (b) scRNA-seq UMAP of integrated FL from control and *Rps19*^*lox/+*^ and *Rpl5*^*lox/+*^ cells with clusters identified by marker genes. (c) Concordance analysis of GSEA pathways between *Rps19*^*lox/+*^ and *Rpl5*^*lox/+*^ models in HSPC, EP and ProE. (d) List of overlapped significantly enriched pathways (FDR < 0.05) highlighted in red in plot in (c). (e) Scatterplots depicting differential expression patterns of the most significantly altered genes between the *Rps19*^*lox/+*^ and *Rpl5*^*lox/+*^ mouse models in HSPC, EP and ProE.

To further explore how the transcriptional landscape is affected during fetal hematopoiesis in *Rps19* and *Rpl5* mutant mice, we performed single-cell RNA-sequencing (scRNAseq) on unfractionated fetal liver cells isolated at E13.5 from two WT and two mutant embryos for each deletion (Fig. 4a). The results were analyzed using an unsupervised clustering approach previously described^33,34^. Cluster identities were assigned based on the expression of the most highly expressed and cluster-specific marker genes, enabling us to distinguish 18 different populations in agreement with published literature^35,36^ (Fig. 4b, **Figure S5a**). Comparison of the clusters between *Rps19*^*lox/+*^ and control littermates based on their gene expression profiles revealed that most alterations were observed in the HSPC, megakaryocyte-erythroid progenitor (MEP), megakaryocyte (Mk) and erythroid progenitor (EP) and proerythroblast (ProE) frequencies (**Figure S5b**). However, the cluster distribution in *Rpl5*^*lox/+*^ was more similar to that of their littermate controls compared to *Rps19*^*lox/+*^. This suggests that the different populations were less affected at the transcriptional level by the loss of one *Rpl5* allele (**Figure S5c**). Because *Rps19*^*lox/+*^ and *Rpl5*^*lox/+*^ mice present with distinct defects in HSPCs and erythropoiesis, we performed a comparative analysis of differentially regulated pathways between the two genotypes in HSPCs, EP and ProE populations. Based on a false discovery rate (FDR) < 0.05, we observed that only one pathway was statistically different at the HSPC stage, being upregulated in RPL5 and downregulated in RPS19. There were no statistically different changes at the EP stage; and most of the changes were observed in the ProE population (Fig. 4c, d). At the individual gene level, *Cdkn1a*, encoding p21, was highly upregulated in HSPCs from *Rps19*^*lox/+*^, along with other genes involved in apoptosis such as *Bax;* in contrast, *Rpl5*^*lox/+*^ exhibited increased expression in the genes encoding the a- and b-globin chains (Fig. 4e). This is consistent with the observation that *Rps19*^*lox/+*^ HSPCs experience deleterious stress and *Rpl5*^*lox/+*^ HSPCs exhibit a greater compensatory response to erythropoietic defects.

Taken together, these data suggest that in addition to defects in translation, RP haploinsufficiency perturbs the transcriptional landscape during fetal hematopoiesis; however, its impact on transcription is specific and depends on the RP subunit affected, being more global in *Rps19*^*lox/+*^ mice than in *Rpl5*^*lox/+*^ mice.

### p53 activation triggers distinct cell death mechanisms in RPS19 and RPL5 haploinsufficient fetal HSPCs.

**Figure 5:**
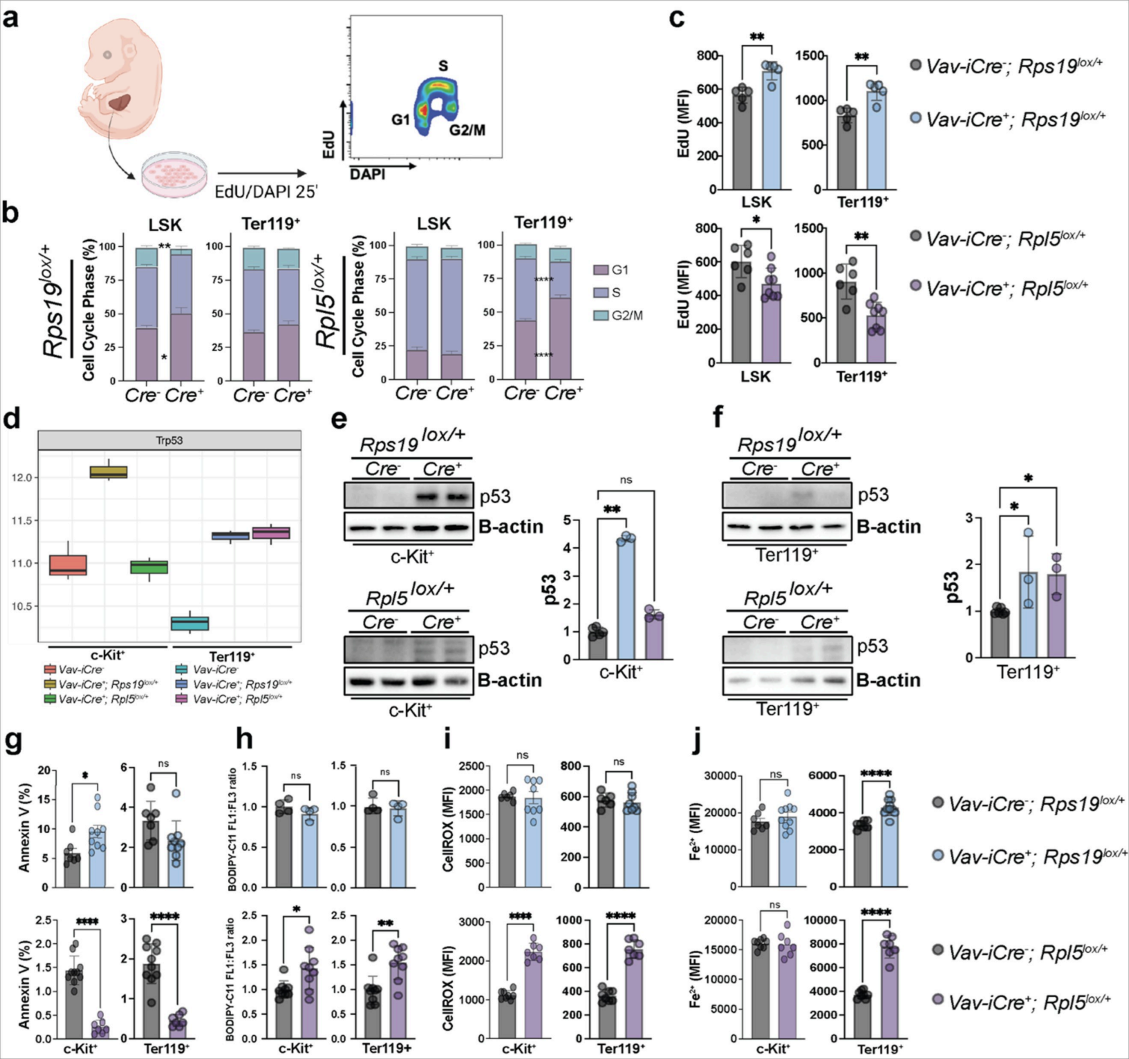
G1 accumulation and p53 activation lead to distinct mechanisms of hematopoietic cell death in *Rps19* and *Rpl5* haploinsufficiency. (a) Experimental design of EdU incorporation in FL cells and cell cycle analysis. (b) G1, S and G2/M phase distribution in LSK and Ter119^+^ populations in E13.5 control, mutant *Rps19*^*lox/+*^ or mutant *Rpl5*^*lox/+*^ embryos. (c) Quantification of the cell cycle speed by measuring the S phase (EdU^+^) MFI among the LSK and Ter119^+^ populations in E13.5 control, *Rps19*^*lox/+*^ or *Rpl5*^*lox/+*^ embryos. (d) Expression levels of *Trp53* in cKit^+^ and Ter119^+^ populations in E15.5 control, *Rps19*^*lox/+*^ or *Rpl5*^*lox/+*^ embryos. (e) Western blot analysis of p53 and △-actin and quantification of p53 normalized to D-actin in FL- derived ckit^+^ cells from E15.5 control versus *Rps19*^*lox/+*^ embryos. (f) Western blot analysis of p53 and D-actin and quantification of p53 normalized to D-actin in FL- derived ckit^+^ cells from E15.5 control versus *Rpl5*^*lox/+*^ embryos. (g) Percentage of Annexin V^+^ cells as marker of apoptosis in E15.5 control, *Rps19*^*lox/+*^ or *Rpl5*^*lox/+*^ embryos. (h) Quantification of cellular ROS levels of cKit^+^ and Ter119^+^ populations as measured by CellROX dye in E15.5 control, *Rps19*^*lox/+*^ or *Rpl5*^*lox/+*^ embryos. (i) Quantification of ferrous iron levels of cKit^+^ and Ter119^+^ populations measured as by Fe^2+^ biotracker dye in E15.5 control, *Rps19*^*lox/+*^ or *Rpl5*^*lox/+*^ embryos. (j) Quantification of cellular lipid peroxidation level of cKit^+^ and Ter119^+^ populations measured by the ratio of oxidized and non-oxidized BODIPY dye in E15.5 control, *Rps19*^*lox/+*^ or *Rpl5*^*lox/+*^ embryos. All data are presented as mean ± standard deviation (*p<0.05, **p<0.01, ***p<0.001, ****p<0.0001).

Having established translational and transcriptional alterations in the RPS19 and RPL5 haploinsufficient mice, we sought to understand the molecular mechanisms leading to the HSPC defects in these models. The loss of HSPCs in RPS19 but not in RPL5 haploinsufficient mice led us to hypothesize that their cell cycle dynamics might be different. To test this, we analyzed the cell cycle characteristics of HSPCs isolated from E13.5 fetal liver cells using EdU and DAPI (Fig. 5a). All *Rps19*^*lox/+*^ progenitors—including Lin- and LSK cells—exhibited G1 accumulation. However, consistent with the accumulation of progenitors in RPL5-haploinsufficient mice, cell cycle entry in *Rpl5*^*lox/+*^ LSK progenitors was maintained. Indeed, in *Rpl5*^*lox/+*^ fetal liver cells, defective cell cycle entry was only detected in the Ter119^+^ compartment (Figs. 5b and S6a). Furthermore, the rate of EdU incorporation during S phase, indicative of S phase speed, was augmented in all *Rps19*^*lox/+*^ progenitors but significantly attenuated in *Rpl5*^*lox/+*^ progenitors (Figs. 5c, d and S6b). These data reveal marked differences in the cell cycle dynamics of RPS19- and RPL5-haploinsufficient fetal progenitors, with the former cycling more rapidly and the latter more slowly.

Current understanding of a principal mechanism by which ribosomopathies disrupt the normal behavior of cells is that activation of nucleolar stress—mediated by accumulation of 5S-RPL11- RPL5 particles—leads to cell cycle block in G1 due to p53 activation, and apoptotic cell death^37^. The differences observed in G1 accumulation at different stages between the two models led us to interrogate the levels of p53 in the HSPC and Ter119^+^ populations. There was a 4-fold increase in the levels of p53 in cKit^+^ HSPC in RPS19 haploinsufficient cells, while p53 in *Rpl5*^*lox/+*^ mice was the same as in WT controls (Fig. 5e, 5f). In contrast, in both RPS19 and RPL5 haploinsufficient Ter119^+^ populations exhibited a comparable and significant increase in p53 expression (Fig. 5e, 5g).

Having demonstrated a block in G1 and increased p53 expression in both models, albeit at different stages of differentiation, we assessed the level of apoptosis in the different HSPC populations. *Rps19*^*lox/+*^ mice presented with significant increases in apoptosis at E15.5— measured as a function of Annexin V expression—in HSPC subsets but not in Ter119^+^ cells (Figs. 5h, S6c) and validating our scRNAseq data, that demonstrated increased expression in genes related to apoptosis (Fig. 4e). Surprisingly, and contrary to previously published studies, all populations of *Rpl5*^*lox/+*^ cells, including Ter119 + erythroblasts, exhibited significantly lower levels of apoptosis (Figs. 5h, S6c). Collectively, these results strongly suggest that the depletion of HSPCs in *Rps19*^*lox/+*^ mice is primarily due to a p53-induced cell cycle arrest and cell death. In contrast, RPL5 haploinsufficiency does not alter cell cycle dynamics in HSPCs. Indeed, these cells exhibit decreased apoptosis relative to their WT counterparts.

As increased p53 expression has been linked to ferroptosis which recently emerged as an alternative mechanism of cell death following ribosomal and oxidative stress^38,39^, we hypothesized that ferroptosis was involved in the loss of *Rpl5*^*lox/+*^ Ter119^+^ cells. Consistent with this, scRNAseq analyses demonstrated an upregulation of pro-ferroptosis genes and downregulation of anti- ferroptosis genes as cells progressed towards erythroid differentiation in RPL5, but not in RPS19 haploinsufficient mice (**Figure S7**). To further assess how these changes in gene expression affected the cells, we assessed oxidative stress, Fe^2+^ accumulation and lipid peroxidation (as measured by the ratio BODIPY C:11/C:13^39^) in *Rps19* and *Rpl5* haploinsufficient mice. While neither oxidative stress nor Fe^2+^ accumulation or lipid peroxidation was augmented in *Rps19*^*lox/+*^ progenitors, they were significantly augmented in Ter119^+^ erythroblasts from *Rpl5*^*lox/+*^ animals (Fig. 5i–k, S6d, e).

Thus, the RPS19 and RPL5 haploinsufficient models exhibit divergent and differentiation stage- specific responses downstream of p53 activation: RPS19-haploinsufficient HSPC undergo apoptosis, whereas RPL5-haploinsufficient EPs undergo ferroptosis resulting from oxidative stress.

### Complete loss of p53 restores HSPC numbers in RPS19 while rescuing erythroid progenitors’ capacity to differentiate in RPL5 haploinsufficient mice.

**Figure 6:**
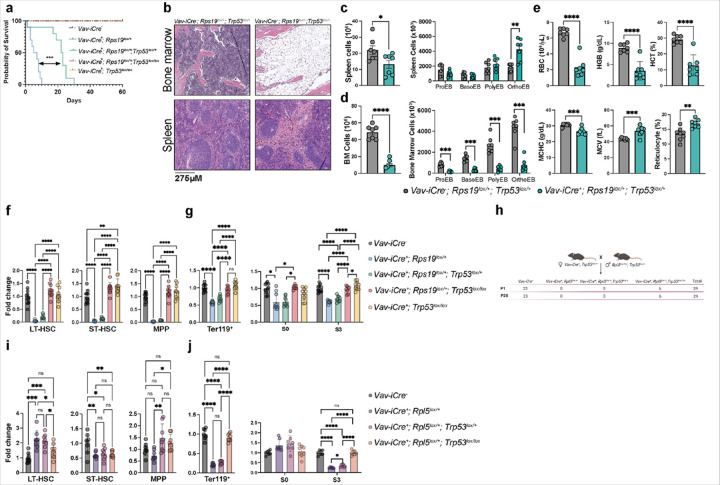
Rescue of the hematopoietic defects in both RP insufficient models require the complete ablation of p53. **(a)** Survival curves from control*, Rps19*^*lox/+*^*, Rps19*^*lox/+*^*; p53*^*lox/+*^*, Rps19*^*lox/+*^*; p53*^*lox/lox*^ and *p53*^*lox/lox*^ mice. **(b)** Light microscopy images highlighting the architecture and cellular composition of the bone marrow (BM) and spleen in control and *Rps19*^*lox/+*^*; p53*^*lox/+*^ mice at P21. **(c)** Spleen cellularity and quantification of terminal erythropoiesis by flow cytometry based on CD44/Ter119/FSC as markers of differentiation in control and *Rps19*^*lox/+*^*; p53*^*lox/+*^ mice at P21. **(d)** BM cellularity and quantification of terminal erythropoiesis by flow cytometry based on CD44/Ter119/FSC as markers of differentiation in control and *Rps19*^*lox/+*^; *p53*^*lox/+*^ mice at P21. **(e)** Red cell parameters at P21. Upper panel: red blood cells (RBC), hemoglobin (HGB), hematocrit (HCT). Lower panel: mean corpuscular hemoglobin concentration (MCHC), mean corpuscular volume (MCV), reticulocytes. **(f)** Quantification of the different HSPC populations in E17.5 control, *Rps19*^*lox/+*^*, Rps19*^*lox/+*^*; Rps19*^*lox/+*^*, Rps19*^*lox/+*^*; p53*^*lox/+*^*, Rps19*^*lox/+*^*; p53*^*lox/lox*^ and *p53*^*lox/lox*^ embryos expressed as a fold change relative to control. **(g)** Ter119+ cell counts and quantification of cells in S0 and S3 in E17.5 control, *Rps19*^*lox/+*^*, Rps19*^*lox/+*^*; p53*^*lox/+*^*, Rps19*^*lox/+*^*; p53*^*lox/lox*^ and *p53*^*lox/lox*^ embryos expressed as a fold change relative to control. **(h)** Genotype counts from *Rpl5*^*lox/+*^; *p53*^*lox/lox*^ intercrossing. **(i)** Quantification of the different HSPC populations in E17.5 control, *Rpl5*^*lox/+*^*, Rpl5*^*lox/+*^*; p53*^*lox/+*^ and *Rpl5*^*lox/+*^*; p53*^*lox/lox*^ embryos expressed as a fold change relative to control. **(j)** Ter119+ counts and quantification of cells in S0 and S3 in E17.5 control, *Rpl5*^*lox/+*^*, Rpl5*^*lox/+*^; *p53*^*lox/+*^ and *Rpl5*^*lox/+*^*; p53*^*lox/lox*^ embryos expressed as a fold change relative to control. All data are presented as mean ± standard deviation (*p<0.05, **p<0.01, ***p<0.001, ****p<0.0001).

To directly assess the contribution of *Trp53* to the hematopoietic failure observed in RPS19 and RPL5 haploinsufficient models, we bred *Vav-Cre; Rps19*^*lox/+*^ and *Vav-Cre; Rpl5*^*lox/+*^ mice to *Trp53*^*fl/fl*^ mice. In the context of RPS19 haploinsufficiency, survival was improved in a dose-dependence manner (Fig. 6a), with the loss of one allele of *Trp53* increasing survival from 10 to 28 days and the loss of both alleles fully rescuing survival. Surprisingly, histological analyses showed that the bone marrow remained hypocellular in the double heterozygous mice, and that the partial rescue could be attributed to splenic stress erythropoiesis (Fig. 6b, c). Indeed, terminal erythroid differentiation in the bone marrow remained significantly impaired (Fig. 6d). In accord with these data, red cell counts were reduced and both the MCV and reticulocyte counts were increased (Fig. 6e). While the recovery was not sustained— mice still died by 4 weeks of age (Fig. 6a), these data highlight a potential role for p53 in stress erythropoiesis. Remarkably, the complete loss of *Trp53* in the *Rps19*^*lox/+*^ mice led to the complete normalization of the numbers of HSPCs (Fig. 6f), and terminal differentiation was improved, ultimately rescuing survival (Fig. 6a, 6g).

In the context of RPL5 haploinsufficiency, in marked contrast to the *Rps19* haploinsufficient model, deletion of one allele of *Trp53* in the *Rpl5*^*lox/+*^ mice did not improve their survival (Fig. 6h), suggesting that, in double heterozygous mice, stress erythropoiesis is not activated or cannot sufficiently compensate for the anemia observed *in utero*. However, deletion of both alleles of *Trp53* rescued their survival (Fig. 6h). Unlike *Rps19* haploinsufficient mice, HSPC numbers remained unaffected by the deletion of *Trp53*, except for the LT-HSC, which were reduced to levels comparable to littermate controls (Fig. 6i). Nevertheless, the complete deletion of *Trp53* in the *Rpl5*^*lox/+*^ model was associated with a complete recovery of the number of Ter119^+^ cells, and improvement of terminal erythroid differentiation, suggesting that loss of *Trp53* led to the rescue of erythropoiesis (Fig. 6j). In aggregate, these data demonstrate that *Trp53* rescues survival through distinct mechanisms in RPS19 and RPL5 haploinsufficient mice.

### RUNX1 levels are increased in RPS19 haploinsufficient mice and patients, and its conditional deletion rescues HSPC numbers.

**Figure 7:**
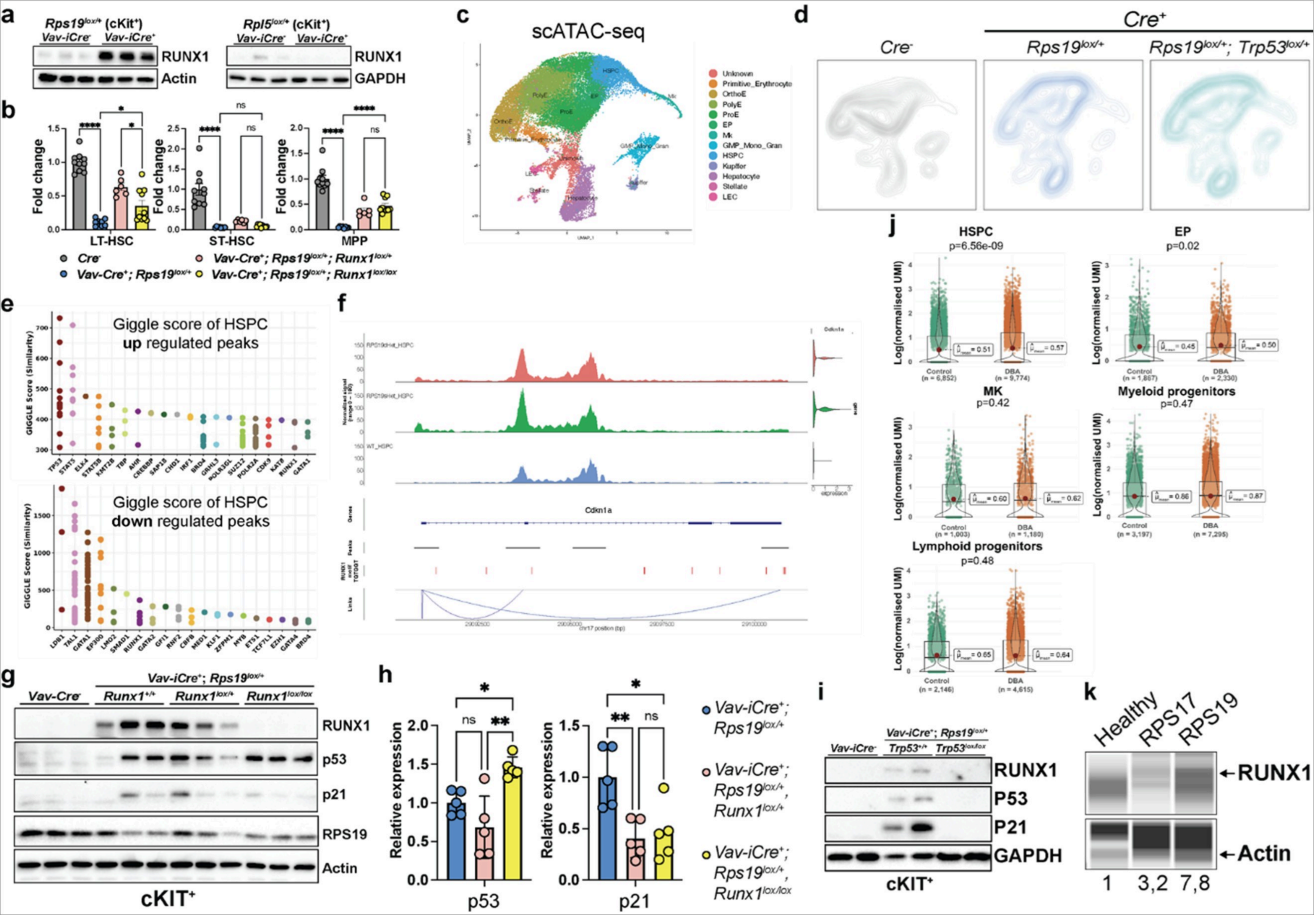
Role of RUNX1 in RPS19 haploinsufficiency. (a) Western blot analyses of RUNX1 expression in ckit^+^ cells from E17.5 control and *Rps19*^*lox/+*^ and *Rpl5*^*lox/+*^ embryos. (b) FL cellularity and quantification of the different HSPC populations in E17.5 control, *Rps19*^*lox/+*^*, Rps19*^*lox/+*^*; Runx1*^*lox/+*^ and *Rps19*^*lox/+*^*; Runx1*^*lox/lox*^ embryos expressed as a fold change relative to control. (c) scATAC-seq UMAP of integrated E13.5 FL from control, *Rps19*^*lox/+*^ and *Rps19*^*lox/+*^*; p53*^*lox/+*^ cells with clusters identified by marker genes. (d) Density projection of cells on scATAC-seq UMAP from control, *Rps19*^*lox/+*^ and *Rps19*^*lox/+*^*; p53*^*lox/+*^ FL cells at E13.5. (e) Differential open chromatin regions are separated into more accessible group (upregulated) and less accessible group (downregulated). The GIGGLE score of 20 predicted transcription factors is displayed in each group. (f) Genome Browser snapshot of the ATAC-seq signal at the *Cdkn1a* gene locus in HSPCs. *Runx1* binding motif (TGTGGT) is highlighted and bottom tracks are analyzed using Signac. (g) Western blot analysis of RUNX1, p53, p21 and RPS19 in FL-derived ckit^+^ cells from E17.5 control, *Rps19*^*lox/+*^, *Rps19*^*lox/+*^; *Runx1*^*lox/+*^ and *Rps19* ^*lox/+*^; *Runx1*^*lox/lox*^ embryos. (h) Quantification of the western blots for p53 and p21 in *Rps19*^*lox/+*^*, Rps19*^*lox/+*^*; Runx1*^*lox/+*^ and *Rps19*^*lox/+*^*; Runx1*^*lox/lox*^ ckit+ cells, normalized to control. (i) Western blot analysis of RUNX1, p53, p21 and RPS19 in FL-derived ckit^+^ cells from E17.5 control, *Rps19*^*lox/+*^, and *Rps19*^*lox/+*^*; p53*^*lox/lox*^ embryos. (j) Violin plots depicting expression of *RUNX1* within stem and progenitor cells from control (n=3) and DBAS (n=6) patients BM, analyzed ex vivo. N refers to total number of cells and the red dot indicates mean expression within each violin. (k) Capillary western blot analysis of RUNX1 and -actin in CD34+ cells from healthy donors and DBAS patients with a mutation in *RPS17* or *RPS19*. All data are presented as mean ± standard deviation (*p<0.05, **p<0.01, ***p<0.001, ****p<0.0001).

Since RPS19 and RPL5 haploinsufficiencies appear to disrupt different signaling pathways downstream of *Trp53* and that its deletion rescues hematopoiesis through different mechanisms, we next sought factors that might explain the divergent phenotypes RPS19 and RPL5 haploinsufficient models. Previous studies have suggested a role for the transcription factor RUNX1 in ribosome biogenesis and translation and have demonstrated reduced p53 activation and apoptosis in *Runx1* knockout HSPCs^40,41^. Further, single-sample GSEA (ssGSEA) from our polysome sequencing experiments showed increased activity in RUNX1 targets involved in HSC differentiation (**Figure S8a**). Interestingly, we observed increased expression of *Runx1* in *Rps19*^*lox/+*^ in HSPC and downstream erythroblast populations while it was decreased in *Rpl5*^*lox/+*^ compared to littermate controls (**Figure S8b**). Furthermore, western blot analysis of c-kit^+^ cells at E15.5 confirmed a striking increase in expression of RUNX1 in *Rps19*^*lox/+*^ compared to littermate controls but not in *Rpl5*^*lox/+*^ embryos, suggesting that *Runx1* does not play a significant role in RPL5 haploinsufficiency (Fig. 7a). Based on these data, we investigated whether knocking out *Runx1* in HSCs would rescue the hematopoietic defects in *Rps19*^*lox/+*^ mice. To this end, we bred *Runx1*^*fl/fl*^ mice to the *Vav-Cre; Rps19*^*lox/+*^ and monitored survival. While double heterozygous mice were born, they did not reach weaning age, and none of the mice in which two copies of *Runx1* had been conditionally deleted in the *Rps19*^*lox/+*^ background were born (**Figure S8c**). We then assayed erythropoiesis, and in the E15.5 embryos we observed that the fetal liver cellularity was unchanged compared to the *Rps19*^*lox/+*^. Similarly, Lin^−^ cells were unaffected by the removal of one or two copies of *Runx1*. Strikingly however, there was a partial rescue of the HSPC compartment, with significant increased numbers of LT-HSC and MPPs (Fig. 7b). Of note, terminal erythropoiesis was significantly worsened in the absence of *Runx1* (**Figure S8d**), suggesting that these mice died of erythropoietic failure.

To understand the relationship between *Trp53* and *Runx1* in the molecular mechanism leading to hematopoietic failure in the *Rps19*^*lox/+*^ animals, we performed combined scRNAseq and scATACseq analyses using the *Vav-Cre*^*+*^*; Rps19*^*lox/+*^*, p53*^*lox/+*^ mice versus controls. We reasoned that since deletion of one allele of *Trp53* did not correct the HSPC defects (Fig. 6), using these animals would limit confounders in a potential role for *Runx1*. UMAP representation of the scRNAseq integrated results, density projection of each individual dataset and GSEA confirmed alterations in the HSPC subsets in the *Vav-Cre*^*+*^*; Rps19*^*lox/+*^ compared to the control littermates (**Figure S9a, b**). The *Rps19*^*lox/+*^*, p53*^*lox/+*^ HSPC presented with significant positive enrichments in GSEA involved in ribosome biogenesis among others (**Figure S9c, d**).

To identify transcription factors that may have contributed to the rescue of the HSPC phenotype in *Rps19*^*lox/+*^*, p53*^*lox/+*^ we characterized regions that underwent chromatin accessibility changes using our scATACseq results from the same three conditions (Fig. 7c, d) using the Cistrome Gene Analysis Toolkit (http://dbtoolkit.cistrome.org/)^42^. We first identified differential open chromatin region (OCR) modules (more accessible or less accessible) in each condition. We then assessed the TF and chromatin regulator enrichment score (Giggle score) in the OCRs, which identifies enriched motifs for known transcriptional regulators by comparing the input file to thousands of reference files from databases such as ENCODE^43^. The Cistrome analysis (Fig. 7e) suggests that regions of GATA1/LDB1/LMO2/TAL1/P300 complex occupancy lose accessibility in *Vav-Cre*^*+*^*; Rps19*^*lox/+*^ HSPCs, which is consistent with the subsequent effect on erythropoiesis. Notably, *Trp53* is the most enriched factor in the more accessible regions as expected. Interestingly, RUNX1 is enriched in the differential OCRs, highlighting the dysregulation of RUNX1 regulatory network in the context of RPS19 haploinsufficiency. Published ChIP-seq data from mouse bone marrow and HSPCs^44,45^ confirmed the binding of RUNX1 on the *Cdkn1a* gene (**Figure S9e**). Furthermore, sc-ATAC results demonstrated increased chromatin accessibility in these RUNX1 binding regions in *Rps19*^*lox/+*^ HSPCs, and no rescue of chromatin accessibility was observed upon p53 haploinsufficiency (Fig. 7f). Taken together, these results demonstrate that RUNX1 plays a role in the mechanism of HSPC failure in RPS19 haploinsufficiency through direct binding to the *Cdkn1a* promoter. We also performed western blot assays on the c-Kit^+^ fraction at E15.5. We observed that while the p21 expression levels were dramatically reduced in the absence of *Runx1*, the levels of p53 remained unchanged (Fig. 7g, h). Conversely, in the *Rps19*^*lox/+*^; *Trp53*^*lox/lox*^ c-Kit + cells, the levels of RUNX1 were back to baseline levels, suggesting that Trp53 is upstream of Runx1 (Fig. 7i). Together, these results indicate that RUNX1 acts between p53 and p21 in the mechanism leading to hematopoietic failure in *Rps19*^*lox/+*^.

Finally, as proof of principle about the direct relevance of these findings to human DBAS, we investigated the expression of RUNX1 in primary bone marrow HSPCs from patients with DBAS by reanalyzing a recently published dataset^46^. This showed a selective increase in *RUNX1* expression in HSPC and EPs but not myeloid or lymphoid progenitors or megakaryocytes in patients with DBAS compared to age-matched controls (Fig. 7j). We further confirmed elevated expression levels of RUNX1 in undifferentiated CD34^+^ cells directly isolated from patients compared to CD34^+^ cells from healthy controls (Fig. 7k). Altogether, these results suggest that RUNX1 plays a role in HSPC depletion in RPS19 haploinsufficiency and that this mechanism may be conserved in patients with DBAS.

## DISCUSSION

Here, we provide a comprehensive evaluation of the role of the ribosomal proteins RPS19 and RPL5 during fetal hematopoiesis in mice with a focus on erythropoiesis and offer a mechanistic explanation at the functional and molecular level for the differences observed in patients with RPS and RPL ribosomopathies. Using two clinically relevant models of DBAS, we demonstrate that while they both have a similar anemic phenotype, the severity and mechanisms are different and distinct depending on the RP subunit deleted. We found that although both RP subunits are critical for fetal hematopoiesis, RPS19 is essential from the HSPC stage while RPL5 appears to be critical after erythroid commitment has occurred. We identified different phenotypes in the HSPC compartments, with progressive depletion in the *Rps19*^*lox/+*^ and expansion in the *Rpl5*^*lox/+*^ animals. These results uncover fundamental differences in the activity of RPS19 or RPL5 in HSPC compared to the more committed erythroid progenitors. They further our understanding of the role of p53 and unravel a potential function for RUNX1 in the mechanisms leading to DBAS.

Focusing on RPS19 and RPL5, as they are the most commonly mutated RPs in ribosomopathies^47^, we demonstrated that the removal of one allele during definitive hematopoiesis leads to severe defects with different consequences based on the lineage affected. The pressure was mostly exerted on the erythroid compartment, eventually leading to death due to anemia. Since the liver is the major site of erythropoiesis in fetal life, potential defect in other hematopoietic lineages may be “masked” by the abundance of erythroid cells. However, our scRNAseq studies showed that the fetal myeloid output was not affected in our models, in accordance with recently published studies demonstrating that fetal HSPCs have diminished steady-state myeloid cell production compared with adult HSPCs^48^. Nevertheless, at birth, we noticed pancytopenia in the *Rps19* model, suggesting that *RPS19* may play different roles in fetal vs adult HSPCs. Whether the same holds true for *Rpl5* remains unknown, since haploinsufficient RPL5 mice die at birth.

Phenotypically, we observed anemia in both models of haploinsufficiency; however, the mechanisms leading to red cell failure are distinct depending on the RP affected. One possible explanation for the phenotypic differences between RPS19 and RPL5 is the mechanism of cell death. Indeed, *Rps19* haploinsufficient HSPCs had increased apoptosis while in Rpl5 haploinsufficient cells, we observed ferroptosis, which was exacerbated as cells differentiated towards the red cell lineage. This model would be consistent with previous studies demonstrating heme imbalance in patients with DBAS where mutations in genes other than RPS19 were involved^49^. This heme imbalance leads to an excess in free heme, which in turn can activate oxidative stress and ferroptosis^38^. Thus, RPL5 haploinsufficiency could result in accumulation of free heme leading to ferroptosis and progressive cell death, an accumulation of cells in S0 (erythroid progenitors), and a block in terminal erythroid differentiation. In contrast, Rps19 haploinsufficiency causes HSPC death by apoptosis leading to progressive depletion of the HSPC compartment. However, cells that are able to reach the erythroid progenitor stage can differentiate up to the S3 (polychromatophilic) stage where a second defect causes severe erythropoietic failure in both models.

Our findings clarify, and reconcile, the role of different factors previously implicated in DBAS. Indeed, studies have proposed a mechanism converging on the defective translation of GATA1 in the erythroid compartment^50^, while others have suggested a role for protein chaperones^49^, heme imbalanced production^51^, or *trp53*^8^ among others. Many of these studies were performed in vitro, using shRNA technologies to knock down RPs or using cells isolated from patients, with the caveat that very few HSPCs could be recovered. Using our *in vivo* models, we demonstrate that indeed, heme synthesis is more affected in RPL5 than in RPS19 haploinsufficient mice and that GATA1 is essential to the defects observed in the erythropoietic failure, although it is not central to the disease, since in the RPS19 haploinsufficient model, the defect originates at a stage prior to GATA1 expression. Our findings complement and expand recent ex vivo studies showing two distinct cellular trajectories segregating with the ribosomal subunit mutated^46^.

These RPS19 and RPL5 haploinsufficiency models are the first authentic models of ribosomopathies that recapitulate more accurately DBAS in humans in which reticulocytosis does not occur, due to the absence of stress erythropoiesis in the spleen. Indeed, in other murine models of bone marrow failure or anemia, extramedullary erythropoiesis occurs in the spleen, compensating for the failure in red cell production in the marrow, and confounding some of the findings^52–54^. To our knowledge, the ISAM mouse, a model of EPO deficiency, is the only model of impaired erythropoiesis without splenomegaly^55^. The absence of stress erythropoiesis enabled us to unravel a role for tp53 in this process with a specificity for the RP deleted. Indeed, the ablation of one allele led to an increase in survival in *Rps19*^*lox/+*^ mice, but not in the *Rpl5*^*lox/+*^ model. Our data demonstrate a rescue of terminal erythroid differentiation in the spleen of *Rps19*^*lox/+*^ pups, partially compensating for the anemia. However, the complete absence of erythropoiesis in the bone marrow points towards different p53-dependent mechanisms in blood cell production depending on the anatomical site. Further, it suggests that stress erythropoiesis in the spleenoriginates from a pool of EPs already present, the so-called stress BFU-E^56^, unlike the marrow pool, which originates from HSC.

With regards to the role of tp53 in ribosomal stress during fetal hematopoiesis, both our models demonstrate increased tp53 expression and its associated targets; however, our polysome- sequencing data in the RPL5 model do not show an increase in the translation of p53, suggesting that the main mechanism at play may reside in its stabilization. We demonstrated that removal of both copies of *Trp53* is necessary for the rescue of the HSPC compartment in the context of RP haploinsufficiency. This suggests that the ribosomal stress experienced by HSPCs is highly sensitive to p53 stability and that its total removal is necessary to bypass it.

We demonstrate that both RPS19 and RPL5 haploinsufficiency cause a decrease in translation in erythroid committed cells. However, this defect is preceded by an increase in protein translation in HSPCs compared to the control littermates, probably contributing to the exhaustion of the HSPC compartment in *Rps19*^*lox/+*^ mice. Although it may seem counterintuitive in the context of RP haploinsufficiency, our data suggest that HSPCs respond to the loss of mature blood cells by increasing differentiation; as this requires more translation^57^, the capacity of the HSPC to maintain protein quality and homeostasis, ultimately leads to HSPC depletion^58^. In contrast, in RPL5 haploinsufficiency, where an increase in translation does not lead to HSPC depletion, there is enhanced translation fidelity, protecting cells from the detrimental effects of increased protein synthesis. Depletion of the HSPC compartment may still occur, but later than in the RPS19 haploinsufficient model. At the molecular level, we identified an increase in mTOR signaling pathway activity, which can compensate for defects in ribosome biogenesis, notably through the phosphorylation of 4E-BP1. Upregulation of 4E-BP1 activity was previously described in the context of Rps6 haploinsufficiency during limb development^59^, but not in the context of hematopoiesis. We further discovered that the activation of 4E-BP1 is stage- and ribosomal protein-specific. Indeed, while 4E-BP1 activity is increased in the c-kit^+^ population of *Rps19*^*lox/+*^ mice compared to control littermates, it goes back to normal levels in the Ter119^+^ population. In contrast, no changes are observed either in c-kit^+^ or in Ter119^+^ cells in the RPL5 haploinsufficient mice. Beyond the mTOR signaling pathway, we demonstrate differential hypusination of eIF5A based on the ribosomal subunit deleted. Along with our previous study highlighting the critical nature of eIF5A-dependent mitochondrial function in regulating the erythroid commitment of HSPCs^27^ and other studies reporting its role in preventing ribosome stalling^28^, our results demonstrate specific metabolic regulations depending on the subunit affected and illustrate the need for further mechanistic studies for therapeutic applications.

Finally, defects at the HSPC level in *Rps19*^*lox/+*^ mice are also seen in *RPS19*^*+/−*^ patients, we hypothesized that another factor may be involved in the defect and identified RUNX1, which plays a key role in developmental hematopoiesis^60^, as a potential candidate. RUNX1 is known to be involved in defective ribosome biogenesis, and its loss of function was linked to decreased translation in HSPCs^40^. We demonstrate using both genetic and functional approaches that RUNX1 is indeed playing a key role in the defects we observe during fetal hematopoiesis and suggest that it could play a role in ribosomopathies independently of tp53. Indeed, when we genetically remove *Runx1* in the *Rps19*^*lox/+*^ model, we observe a normalization of the expression levels of p21, while the levels of p53 remain elevated. While the removal of RUNX1 does not rescue erythropoiesis or survival in the RPS19 haploinsufficient mice, it partially rescues the numbers of HSPCs. This finding suggests a role for RUNX1 in the cancer predisposition observed in patients with ribosomopathies. Indeed, recent reports have demonstrated that patients with a mutation or a deletion in a ribosomal protein have a 4- to 5-fold risk of developing cancer^61^. However, the mechanisms leading from a hypoproliferative to a hyperproliferative condition, also known as the Dameshek’s riddle remain unknown^62^. We suggest that the increased expression of RUNX1 could play a role in the mechanism leading to tumorigenesis. In support of this hypothesis, we observe elevated c-Myc signatures by polysome-sequencing in our models of ribosomal protein haploinsufficiency. Along with the role of p53 in ribosomopathies, this finding opens a new field of investigation for understanding cancer predisposition in hematopoietic disorders.

## Limitations of the study

Our study unravels the roles of RPS19 and RPL5 during fetal hematopoiesis and the impact of removing one allele on the hematopoietic compartment during definitive hematopoiesis. While it expands on findings from other RP deleted in other tissues or during adult hematopoiesis, it will be essential to examine whether our results can be extrapolated to other RP and if observed defects segregate according to whether RP in the small versus large ribosomal subunits are affected. Further, due to the limited number of HSPC, polysome profiling and sequencing experiments were difficult to perform on isolated populations beyond the cKit^+^ population. Finally, while we validated the increased expression of RUNX1 in patients with DBAS, additional studies are required to explore its role during human hematopoiesis in the context of ribosomal protein haploinsufficiency.

## Supplementary Files

This is a list of supplementary files associated with this preprint. Click to download.

- TableS1.docx
- Figures.docx
- METHODS.docx
